# Performance of large language model in cross-specialty medical scenarios

**DOI:** 10.1186/s12967-025-07577-x

**Published:** 2025-12-22

**Authors:** Zhen Cui, Wuzheng Liu, Xuan Tian, Conglei You, Xiangyu Meng, Huijuan Zhang, Kangzi Gong, Xu Wang, Jun Wu

**Affiliations:** 1 Department of Clinical Laboratory, Beijing Jishuitan Hospital, Capital Medical University, Xicheng District, Beijing, 100035 China; 2 Department of Vascular Surgery, Beijing Jishuitan Hospital, Capital Medical University, Xicheng District, Beijing, 100035 China; 3 Health Management Center, Beijing Jishuitan Hospital, Capital Medical University, Xicheng District, Beijing, 100035 China; 4 Department of Central Laboratory, Beijing Jishuitan Hospital, Capital Medical University, Xicheng District, Beijing, 100035 China; 5 Department of Rheumatology and Immunology, Beijing Jishuitan Hospital, Capital Medical University, Xicheng District, Beijing, 100035 China; 6 Department of Obstetrics and Gynecology, Beijing Jishuitan Hospital, Capital Medical University, Xicheng District, Beijing, 100035 China

**Keywords:** Large language model, Artificial intelligence, Diagnosis, Therapeutic recommendations

## Abstract

**Background:**

Large language models (LLMs) demonstrate transformative potential in healthcare, yet their diagnostic and therapeutic accuracy across medical specialties remains inadequately characterized.

**Methods:**

This study aimed to compare diagnostic and therapeutic capabilities of GPT-4o, GPT-3.5-Turbo, Claude-3-Sonnet across 12 medical specialties using standardized clinical vignettes. 50 PubMed-derived clinical cases between 2007 and 2024 were assessed. Two board-certified physicians independently evaluated LLMs outputs, with a senior clinician adjudicating discrepancies. All LLMs received identical text-based case descriptions with or without images, generating free-text diagnostic and therapeutic recommendations for blinded, randomized evaluation.

**Results:**

Among the three evaluated LLMs, GPT-4o demonstrated superior diagnostic accuracy (median 10; IQR, 7.5–10), outperforming Claude-3-Sonnet (median 8; IQR, 2.8–10; *P* = .02) and GPT-3.5-Turbo (median 4; IQR, 1–9.3; *P* < .0001). A narrow IQR and minimal variation (SD = 2.9; range = 5.0) reflected high consistency in diagnostic outputs across diverse medical fields. For therapeutic recommendations, GPT-4o (median 10, IQR 0–10) outperformed GPT-3.5-Turbo (median 0, IQR 0–6.3; *P* = .0005) but showed no significant advantage over Claude-3-Sonnet (median 5, IQR 0–10; *P* = .45).

**Conclusion:**

This study demonstrates that advanced LLMs, particularly GPT-4o, have significant potential to support clinical diagnostics, showing high accuracy and consistency across specialties. However, their inconsistent performance in generating therapeutic recommendations presents a major barrier to clinical adoption.

**Supplementary Information:**

The online version contains supplementary material available at 10.1186/s12967-025-07577-x.

## Introduction

Large Language Models (LLMs) are proving transformative across sectors, and healthcare is embracing them for everything from diagnostic support to personalizing treatments [1]. Among them, OpenAI’s ChatGPT (November, 2022) and Anthropic’s Claude (March, 2023) have emerged as leading candidates for clinical integration due to their capacity to process multimodal data, such as text, images, and longitudinal records [2–4]. Preliminary evidence suggests model-specific strengths: GPT-4o outperforms peers in deductive reasoning (e.g., mathematics and programming), while Claude-3.5-Sonnet excels in biomedical domains such as biochemistry and molecular biology [5]. But critical uncertainties persist regarding how well these models perform across different medical fields, especially when it comes to both diagnostic and therapeutic conditions.

Traditional clinical practices face inherent limitations: variability in clinician expertise, challenges in synthesizing heterogeneous patient data (e.g., medical images, longitudinal records), and delays in aligning decisions with evolving clinical guidelines. These gaps often lead to suboptimal outcomes, underscoring the need for tools that enhance, rather than replace, human judgment. LLMs offer potential by integrating complex information and generating evidence-based recommendations [6], yet their performance across medical specialties and in both diagnostic and therapeutic tasks remains poorly characterized [7].

Most current LLMs research focuses on constrained tasks (e.g., multiple-choice examinations) or theoretical knowledge assessments, overlooking critical clinical challenges such as reconciling contradictory symptoms, interpreting longitudinal patient data, or generating reliable management recommendations [8–12]. Rigorous, comparative evaluations of LLMs (e.g., GPT-4o, GPT-3.5-Turbo, and Claude-3-Sonnet) in simulating clinician-level decision-making are also lacking, limiting evidence-based guidance for clinical model selection.

To address these gaps, we conducted a study comparing three LLMs across 12 medical specialties. Whereas prior studies that primarily focused on the performance of LLMs in a single medical specialty (e.g., radiology or oncology) [13]. By assessing three LLMs across 12 distinct medical specialties, we overcome the narrow scope of prior single-specialty assessments. This comprehensive approach enables a more nuanced understanding of how different LLMs perform in diverse clinical contexts, revealing their strengths and weaknesses across specialties [8–10]. In contrast to prior studies that relied on simplified or standardized case summaries, our free-text vignettes integrate medical images and longitudinal records, mirroring the heterogeneous data encountered in daily clinical practice. This not only more accurately reflects the challenges faced by clinicians in practice but also provides a critical step toward validating their utility in real-world clinical workflows.

This study aims to provide actionable insights for clinicians and developers on LLMs selection and optimization across specialties, ultimately supporting their safe and effective integration into clinical decision-making, particularly in diagnostic and therapeutic conditions.

## Methods

### Case selection and data preparation

**Fig. 1:**
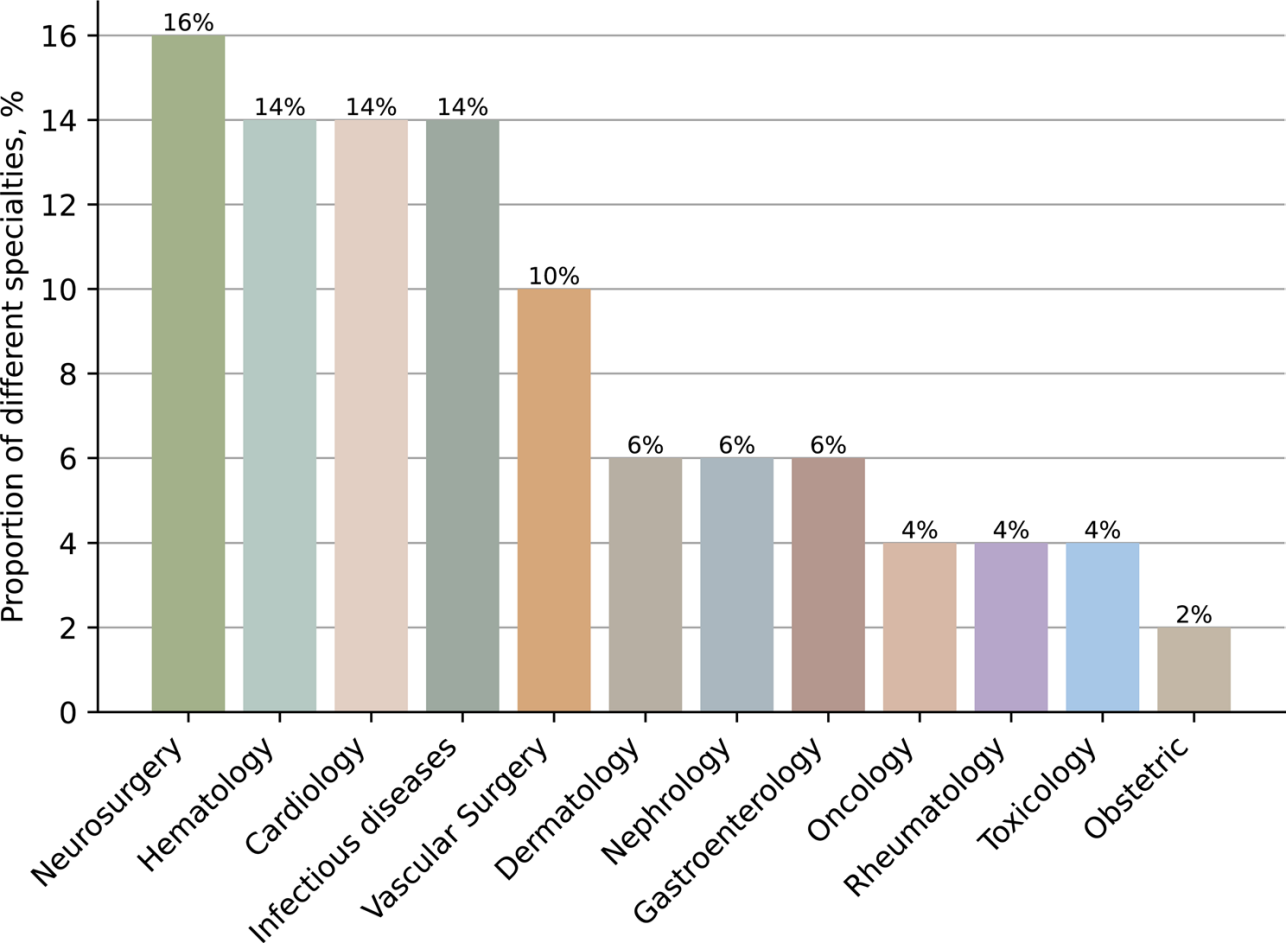
Distribution of clinical cases across medical specialties. By searching the database, we collected 50 clinical cases. These cases are diverse, covering Dermatology, Vascular Surgery, Hematology, Cardiology, Infectious disease, Neurosurgery, Gastroenterology, Obstetric, Oncology, Nephrology, Rheumatology, Toxicology

A total of 50 clinical cases spanning 12 specialties (e.g., Dermatology, Vascular Surgery, Hematology, Cardiology, Infectious Diseases, Neurosurgery, Gastroenterology, Obstetrics, Oncology, Nephrology, Rheumatology, and Toxicology) were systematically retrieved from PubMed (2007–2024). Cases were randomly selected to and included complete clinical narratives (chief complaint, medical history, physical examination, laboratory results) and, where available, medical images. Exclusions included cases with incomplete data or rare diseases (prevalence < 1:10,000). The distribution of cases across medical specialties is illustrated in Fig. 1.

### Large language models (LLMs) prompting protocol

**Fig. 2:**
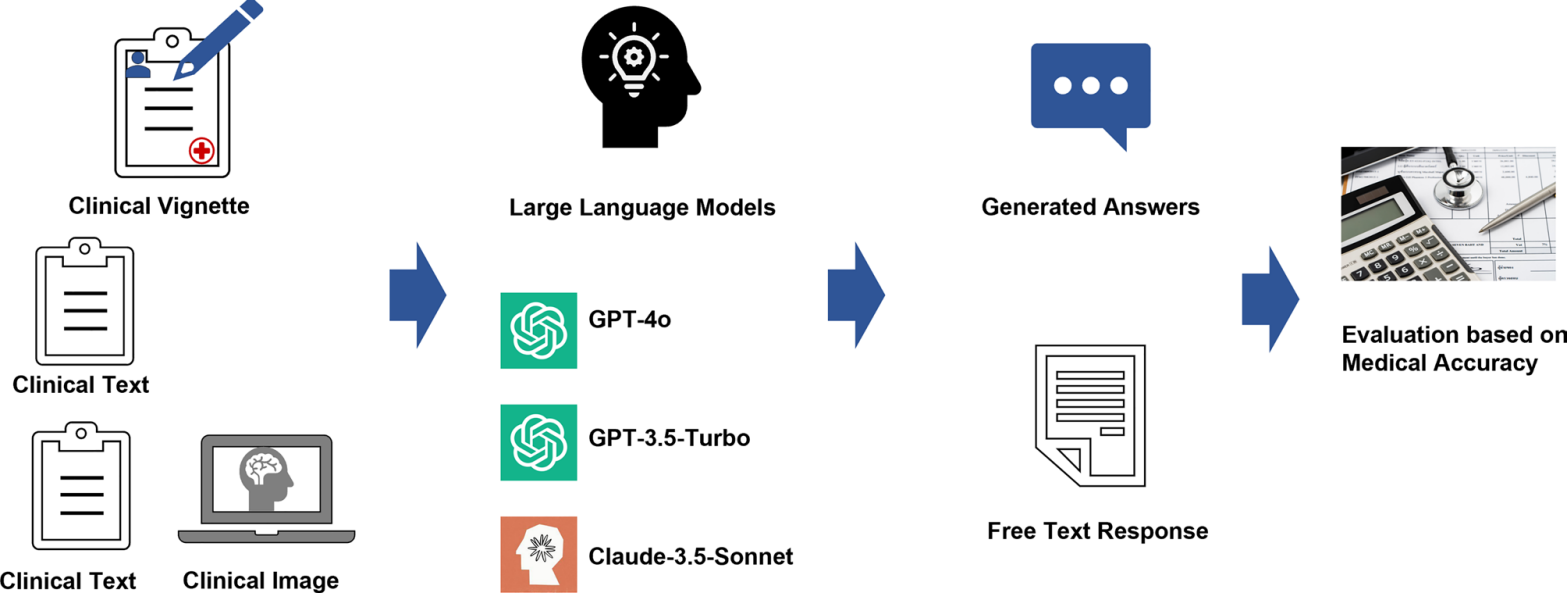
Procedure for LLMs Prompting

Three LLMs were evaluated: GPT-4o (May 2024; OpenAI) [14], GPT-3.5-Turbo (August 2023; OpenAI) [15], and Claude-3-Sonnet (June 2024; Anthropic) [16]. A new chatbot session was initiated for each case to prevent data contamination. The prompting workflow is fully outlined in Fig. 2. The models received standardized inputs (clinical text plus images, when available) with the following instructions: “*Based on the provided patient information*,* and medical history*,* generate a precise diagnostic analysis. Focus on the most likely diagnosis with evidence-based rationale. Provide detailed*,* clinically actionable explanations*.”

### Evaluation framework

A panel of clinicians evaluated responses generated by LLMs using a predefined 10-point rubric with four core dimensions on diagnosis:

1. Diagnostic accuracy (0–10 points): Awarded 10 points if the answer aligns with the diagnosis; Partially correct diagnosis will be scored separately based on criteria 2, 3, and 4 below; otherwise, 0 points.
  10: Diagnosis fully aligned with ground-truth diagnosis and supporting rationale.
  5: Partially correct diagnosis with incomplete justification.
  0: Incorrect diagnosis or no response.
2. Evidence-based reasoning (0–2 points).
  2: Logically sequenced reasoning without contradictions.
  1: Minor inconsistencies in diagnostic logic.
  0: Illogical or internally conflicting arguments.
3. Clinical coverage (0–2 points).
  2: Comprehensive addressing of all critical features (e.g., red flags, comorbidities).
  1: Major omissions in contextual factors.
  0: Superficial or fragmented analysis.
4. Relevance to case data (0–2 points).
  2: Clarity of rationale linking findings to diagnosis.
  1: Partial connection to case data.
  0: Unrelated or irrelevant diagnosis.

There was one dimension on treatment recommendations: Guideline adherence (0–10 points: awarded 10 points if the answer complies with the treatment recommendations; awarded 5 points for partially correct answers; otherwise, 0 points).

Two independent board-certified clinicians scored responses. Incorrect or absent answers received 0 points; answers that were partially correct were scored according to the criteria detailed above. Conflicts were resolved via discussion with a third senior clinician to achieve consensus decision on the accuracy of the response. All responses were anonymized and randomized before evaluation. We calculated a weighted Cohen κ value to show concordance in grading [17]. Prompts and responses related to a clinical case are presented in Table S1 (Supplement Material 1), and a representative clinical case of low-scoring LLMs’ responses to therapeutic recommendations is presented in Table S2 (Supplement Material 1).

### Statistical analysis

Statistical analyses were conducted using Python 3.9.16, and GraphPad Prism 8.0. The Mann-Whitney U test with Bonferroni correction for multiple comparisons was applied to comprehensively assess performance differences, with significance set at *P* < .05 after multiplicity adjustment.

## Results

### LLMs performance in clinical scenarios

A total of 50 clinical cases encompassing diverse conditions were analyzed (Table 1). The weighted Cohen κ value between 2 raters was 0.9, indicating substantial agreement within the expected range for diagnostic performance studies [18]. In diagnostic performance, GPT-4o achieved a median score of 10 (IQR 7.5–10), outperforming Claude-3-Sonnet (median 8; IQR 2.8–10; *P* = .02), and GPT-3.5-Turbo (median 4; IQR, 1–9.3; *P* < .0001). The narrow IQR of GPT-4o indicated high consistency in diagnostic outputs, a critical feature for clinical adoption—consistent results reduce the risk of misdiagnosis in real-world settings where variability can compromise patient outcomes.

**Table 1:** Comparison of the overall diagnostic and treatment responses among the LLMs

LLMs	Median Score (IQR)	95%CI	SD	*P* value	*P* value
**Diagnostic Performance**					
GPT-4o	10 [7.5–10]	7.5–9.2	2.9	.02^a^	< .001^b^
Claude-3-Sonnet	8 [2.8–10]	5.5–7.6	3.7	NA	NA
GPT-3.5-Turbo	4 [1–9.3]	3.9–6.1	3.8	NA	NA
**Treatment Performance**					
GPT-4o	10 [0–10]	5.4–7.8	4.3	.45^c^	.0005^d^
Claude-3-Sonnet	5 [0–10]	4.1–6.5	4.3	NA	NA
GPT-3.5-Turbo	0 [0–6.3]	2–4.4	4.3	NA	NA

Abbreviation: LLMs, large language models, SD, standard deviation. a and c indicate GPT-4o vs. Claude-3-Sonnet, b and d indicate GPT-4o vs. GPT-3.5-Turbo. P values were calculated using Mann-Whitney U with Bonferroni correction for multiple comparisons

For therapeutic recommendations, the weighted Cohen κ value between 2 raters was 0.8, also indicating considerable concordance within the expected range [18]. GPT-4o (median 10; IQR, 0–10) shown no significant advantage over Claude-3-Sonnet (median 5; IQR, 0–10; *P* = .45), despite outperforming GPT-3.5-Turbo (median 0; IQR, 0–6.3; *P* = .0005). The consistent high performance of GPT-4o across both diagnostic and therapeutic applications, especially its robust diagnostic capabilities, strongly supports its viability as a medical assistant analytical tool. Based on a preliminary evaluation of a small-scale, scenario-specific set of cases, our study found that GPT-4o demonstrates a certain potential for assistance, as its performance across medical specialties shows better stability in therapeutic conditions. Conversely, the equivocal results obtained with Claude-3-Sonnet underscore the necessity for extensive validation across diverse clinical settings to precisely delineate its context-dependent efficacy.

### Diagnostic performance disparities

**Fig. 3:**
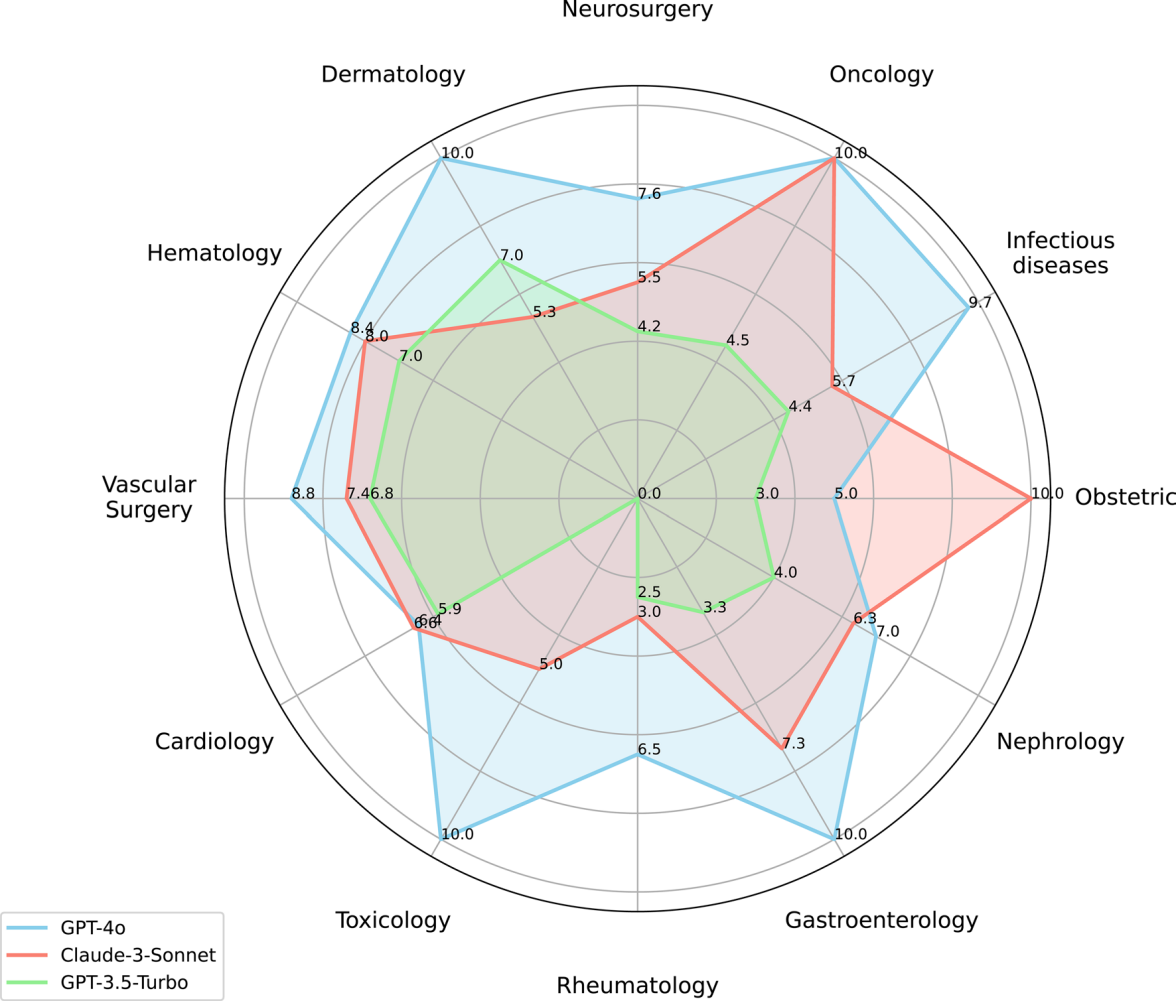
Diagnostic Performance of GPT-4o, Claude-3-Sonnet, and GPT-3.5-Turbo in medical cases from 12 specialties. LLMs, large language models. The GPT-4o, Claude-3-Sonnet, and GPT-3.5-Turbo are noted in blue, purple, and green color respectively. The number represents the average score the model tests for the disease (*N* = 50)

As visualized in the radar chart (Fig. 3), the radial axes represent each specialty, with distance from the center corresponding to performance scores (0–10, 10 = highest). GPT-4o achieved the maximum score (10.0) in four key specialties - Dermatology, Oncology, Toxicology, and Gastroenterology) - and exceeded 8.4 in seven additional domains (e.g., Infectious Diseases, 9.7; Vascular Surgery, 8.8). This minimal variation (SD = 2.9; range = 5.0) in performance across diverse medical fields is reflected in the compact shape of its profile on the chart (Fig. 3) and demonstrates greater consistency compared to competitors, which exhibited wider performance ranges.

Claude-3-Sonnet demonstrated exceptional performance in niche domains, matching GPT-4o’s score of 10.0 in both Oncology and Obstetrics. However, its performance exhibited high variability across specialties (SD = 3.7; range = 7.0), achieving a maximum score of 10.0 but dropping to a minimum of 3.0 in Rheumatology. This significant domain-dependent inconsistency limits its overall reliability compared to the more uniformly high-performing GPT-4o.

GPT-3.5-Turbo achieved moderate scores in select specialties (e.g., 7.0 in Dermatology and Hematology) but scored below 4.5 in the other 8 specialties (e.g., 2.5 in Rheumatology and 4.2 in Neurosurgery). It also exhibited broader dispersion in scores (SD = 3.8; range = 4.5) compared to GPT-4o. This wider variability positions GPT-3.5-Turbo as the least competitive model in this comprehensive medical domain evaluation.

### Therapeutic recommendation performance

**Fig. 4:**
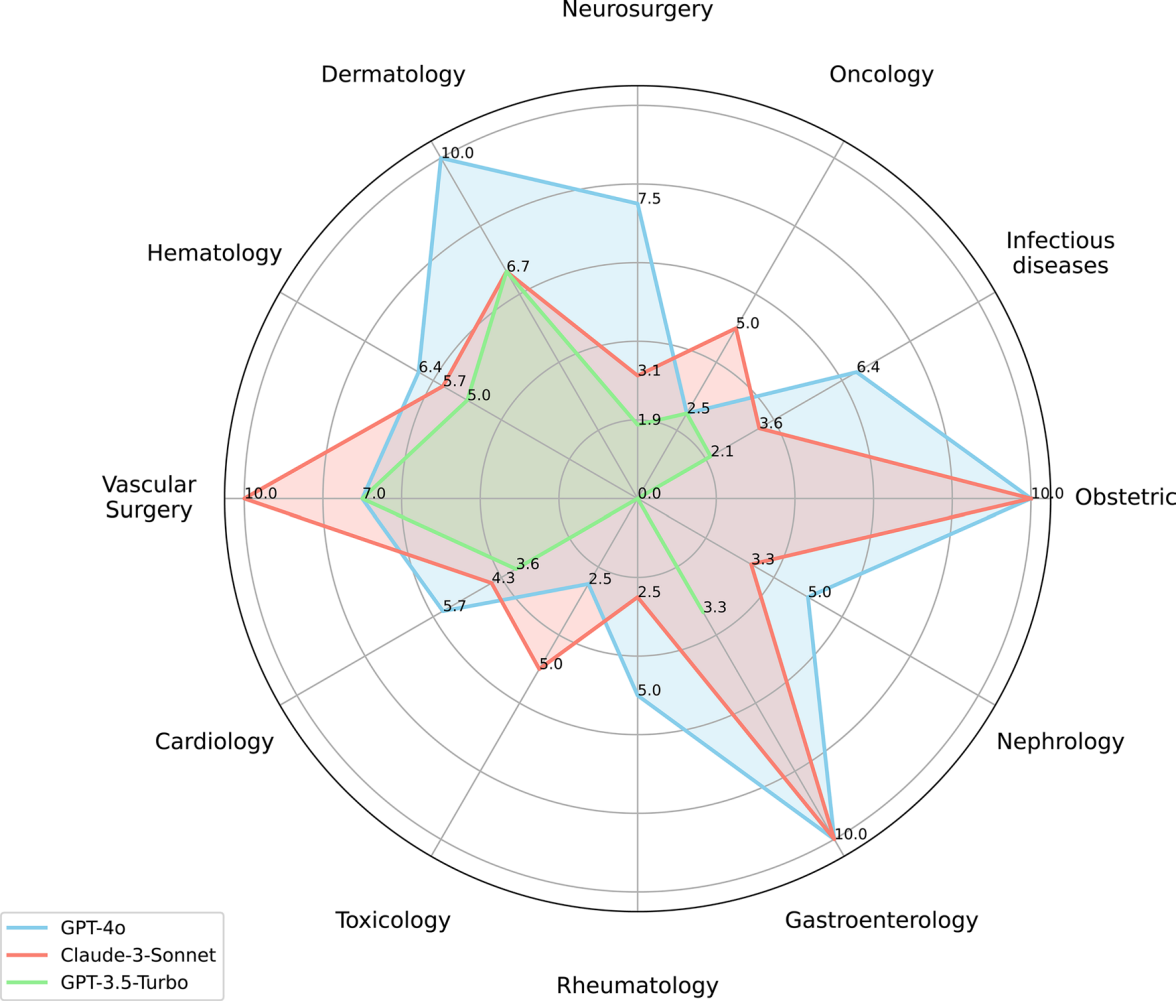
Cross-specialty accuracy of GPT-4o, Claude-3-Sonnet, and GPT-3.5-Turbo in Therapeutic Regimen. LLMs. large language models. The GPT-4o, Claude-3-Sonnet, and GPT-3.5-Turbo are noted in blue, purple, and green color respectively. The number represents the average score the model tests for the disease, (*N* = 50)

It is interesting to note that when provided with diagnostic commands, almost all responses from the LLMs include corresponding treatments, but accuracy varied (Fig. 4). GPT-4o achieved the highest scores (10.0) in Dermatology, Obstetrics, and Gastroenterology. It maintained comparatively strong performance in Vascular Surgery (7.0), Neurosurgery (7.5), and Infectious Diseases (6.4), demonstrating broad competency across specialties. Claude-3-Sonnet excelled in Vascular Surgery (10.0) and matched GPT-4o’s high scores in Obstetrics and Gastroenterology, yet its overall performance was more erratic, with a notably weaker showing in Rheumatology (2.5). GPT-3.5-Turbo scored poorly across all specialties, peaking at 7.0 in Vascular Surgery and never reaching above 5.0 in the other 10 specialties.

GPT-4o showed particular proficiency in generating therapeutic recommendations compared to the other models. A closer examination of its performance reveals a significant vulnerability, with the lowest average scores occurring in two specialties - Oncology (2.5) and Toxicology (2.5). In addition, the errors in GPT-4o’s therapeutic recommendations were thoroughly classified into three categories: incomplete or unreasonable treatment plans (11 cases), failure to perform necessary steps (6 cases), and incorrect medication administration (4 cases). We report the distribution of errors across these categories to highlight priority safety concerns.

## Discussion

This cross-specialty study reveals notable differences in LLMs clinical performance across diagnostic and therapeutic conditions. Consistent with LLMs evolution trends, diagnostic accuracy has incrementally improved in newer models [13]. Our findings confirm this progression, with GPT-4o surpassing GPT-3.5-Turbo and Claude-3-Sonnet in most of the evaluated clinical specialties, underscoring the need for continuous optimization of medical LLMs throughout the clinical decision-making continuum.

Several studies have evaluated the core medical applications of LLMs, yet most remain highly specialized and often address diagnosis and treatment as separate tasks. Many have focused on individual medical specialties, such as clinical chemistry and laboratory management [19], spine surgery [4], radiology quizzes [20] or radiology cases [13], oncology examinations [9] or oncology cases [21], immuno-oncology questions [22], dermatology questions [23, 24], obstetrics and gynecology [25], neurological examinations [26], and generative tasks in mental health care [27]. In addition, comparative analyses of therapy recommendations [28] or treatment plans [29] across various clinical specialties have also been conducted. Our study offers a comprehensive evaluation of clinical applications—encompassing both diagnosis and treatment—and further extends this evaluation to cases spanning 12 clinical specialties. This, in turn, supports LLMs in developing a more holistic understanding of real-world clinical scenarios.

GPT-4o displayed exceptional diagnostic stability (SD = 2.9; range = 5.0), indicating stable knowledge integration across domains. Its high scores in Dermatology, Oncology, Toxicology, and Gastroenterology align with documented strengths in visually-oriented and protocol-driven specialties [19, 23, 30, 31], stands in stark contrast to Claude-3-Sonnet’s notable weakness in Rheumatology (score 3.0). This disparity aligns with prior findings that LLM efficacy is shaped by domain-specific training data quality and architecture complexity [28, 32]. Claude-3-Sonnet’s strength in Obstetrics likely reflects its training on ACOG guidelines, while GPT-4o’s proficiency in Oncology may stem from robust integration of genetic and imaging data. These findings also echoes prior work highlighting the challenges of integrating heterogeneous medical data [33].

Despite diagnostic competence, making appropriate decisions remains difficult [34, 35]. GPT-4o’s therapeutic recommendations exhibit a dramatic interquartile range (IQR 0–10), indicating potential inconsistency, despite having a high median. Notably, one of the central objectives of our research is to quantify such inconsistency, rather than merely reporting the LLMs’ performance. This finding, in turn, provides the strongest evidence for our proposed Human-in-the-loop framework for clinical integration. The wide IQR underscores the urgent need for implementing multi-layered safeguards, including: mandatory expert oversight, all LLMs-generated output must be reviewed and approved by a clinical professional, who serves as the final decision-maker; context-specific restrictions, the model should be strictly limited to low-risk use cases such as knowledge retrieval, differential diagnosis generation, and documentation assistance; uncertainty indication, future clinical LLMs should be equipped with a confidence indication function, issuing clear alerts to users when confidence is low (e.g., in cases falling within the lower quartile observed in this study).

The inconsistency in LLMs-generated therapeutic recommendations stems from a complex interplay of knowledge cutoff and static guidelines, inherent complexity of treatment decisions, and prompting strategy.

Knowledge Cutoff and Static Guidelines. The most significant constraint is the LLMs’ inherent lack of real-time, dynamic knowledge integration. Unlike clinicians who actively consult continuously updated resources like UpToDate [36] or incorporate findings from recent conferences, general-purpose LLMs are trained on static datasets with a fixed knowledge cutoff (e.g., GPT-4o’s training data ends in June 2024). This renders them incapable of incorporating: recent drug approvals and institution-specific protocols; emerging clinical trial evidence; local formulary and protocol guidance.

Inherent Complexity of Clinical Decision-Making. Therapeutic decision-making is a higher-dimensional challenge than diagnostic reasoning. Diagnosis focuses on converging evidence towards a pathophysiological state, but treatment requires balancing patient-specific variables, many of which are poorly captured in text-based prompts. Without access to structured, granular patient data, LLMs inevitably oversimplify this complexity.

Prompting Strategy. Our experimental design, with the prompt “generate a precise diagnostic analysis and treatment recommendation”, may have inadvertently contributed to inconsistent treatment outputs. LLMs are highly sensitive to prompt structure: for example, a prompt that prioritizes “diagnostic analysis” may direct computational resources toward diagnosis, rendering treatment recommendations secondary and less rigorously validated. Rather than performing a deep, patient-specific review of therapeutic options, LLMs may prioritize syntactic coherence (e.g., ensuring the treatment sounds appropriate for the diagnosis) over clinical accuracy.

Within this benchmark evaluation, GPT-4o attained the highest scores while maintaining the smallest performance variation across specialties. Although Claude-3-Sonnet achieved comparable results to GPT-4o in two specialties, it demonstrated the most substantial performance fluctuations among the evaluated systems. GPT-3.5-Turbo yielded inferior results relative to the other two models in twelve specialties. These findings reveal fundamentally distinct competency patterns for medical knowledge application within our assessment framework, highlighting the need to integrate dynamic guidelines and to engineer prompts (e.g., zero-shot chain-of-thought) to enhance contextual alignment [37, 38].

An unexpected finding was that cases with medical images elicited less detailed reasoning than text-only cases, mirroring Chen et al.’s report of reduced multimodal accuracy in image-rich datasets [21]. This suggests that these models have significant shortcomings in combining radiological evidence with clinical narratives, which could hinder their performance in specialties that rely heavily on imaging, such as Cardiology (with echocardiography) and Dermatology (with lesion analysis).

LLMs have demonstrated transformative potential for clinical tasks, yet the current regulatory framework governing their use in healthcare remains inadequate [39]. Concerns around deploying LLMs in clinical practice include ethical implications, safety concerns, and governance accountability.

Ethical Implications. Ethical concerns revolve around the privacy and security of patient data that are used to train these LLMs. As these models rely on vast amounts of personal health information, ensuring the robustness of their data protection processes becomes an essential part of maintaining patient trust and complying with regulatory standards [40]. Processing sensitive health data (e.g., genetic information) violates regulations like the U.S. Health Insurance Portability and Accountability Act (HIPAA) unless stringent safeguards (e.g., federated learning) are implemented [41]. Healthcare data must be securely and confidentially managed, safeguarding both patients from potential harm and healthcare providers from legal liabilities.

Safety Concerns. LLMs may produce responses that are inaccurate, outdated, or entirely fictionalized, a phenomenon known as “artificial hallucination” [42]. Such undesired behavior can negatively impact human-LLMs interaction, undermine trust in AI systems and LLMs, and at worst put people’s health at risk [43]. Importantly, LLMs-generated texts are a reflection of their training data and thus could perpetuate biases pertaining to age, race, sex, language, culture, disease severity and record repetition [33, 44]. This issue poses a risk of influencing clinical decisions and patient care, disproportionately impacting underrepresented groups through biased diagnosis or treatment recommendations [42]. Thus, it is crucial to address this consensus in LLMs to ensure accurate and fair diagnoses.

Governance Accountability. There are well-known challenges in assigning responsibility for errors made by LLMs, and LLMs cannot assess the quality of information they use or produce [45]. It is unclear whether clinicians, institutions, or model developers bear liability when LLMs-driven recommendations result in harm [46], posing legal risks to both clinicians and developers [47]. This highlights the need for further improvements in the accuracy of LLM-generated texts.

Our findings emphasize the importance of specialty-targeted fine-tuning in LLMs. For instance, optimizing LLMs for toxidrome patterns could improve Toxicology performance, while integrating real-time fetal monitoring data may enhance Obstetrics outcomes. When deploying LLMs clinically, rigorous evaluation protocols, including “new chat” resets to mitigate in-context biases [48], clear accountability frameworks for erroneous recommendations [22, 37] and developing hybrid architectures that can incorporate updated guidelines, are imperative.

Further research is warranted to optimize specialty-specific models, integrate real-time guidelines, and rigorously assess their potential to support clinical decision-making within clinician-AI collaborative workflows.

### Limitations

This study used 50 clinical vignettes across 12 specialties, allowing for a broad sampling of different disciplines. The sample of case scenarios used in this study is small and fail to cover key medical scenarios such as complex clinical decision-making, cross-departmental difficult cases, and emergency medical management, leading to significant limitations in the generalizability of the conclusions. The 12 included specialties (Dermatology, Vascular Surgery, Hematology, Cardiology, etc.) are core fields with high potential for LLMs application in clinical practice and significant disparities in medical knowledge systems. All cases in this study were randomly selected through PubMed and are real-world clinical cases, which can reflect the actual performance differences of LLMs across different specialty scenarios. This design not only ensures case quality but also provides key support for the rationale of our current sample. However, the uneven case distribution across the 12 specialties stemming from the limited total sample size gives rise to two potential biases: specialty-specific sampling bias, and confounding bias arising from the interplay between specialty-specific attributes (e.g., diagnostic paradigms, treatment workflows) and disease complexity. No sample of clinical vignettes can fully cover the diverse spectrum of medical cases encountered in practice [49]. All findings of this study are preliminary exploratory results, which only reflect GPT-4o’s performance in specific small-scale scenarios and cannot be extended to broader medical contexts.

Notably, the limitations of literature-derived cases, all of which were retrieved from PubMed literature, may prevent full evaluation of LLMs’ performance in mild cases and dynamic decision-making scenarios. Accordingly, we framed this as a key study limitation to avoid overgeneralizing the conclusions’ applicability. Literature (e.g., case reports, case series) indexed in PubMed tends to prioritize reporting typical cases, and those with special clinical outcomes [50], and it omits critical dynamic information for real-world decision-making: detailed physical signs (e.g., heart rhythm changes), in-treatment adjustments (e.g., antibiotic switches for poor response), and concurrent medications (e.g., liver/kidney-impacting drugs).

Despite the above limitations, the selection of PubMed literature-derived cases in this study is a trade-off. The main reasons are as follows: first, considerations regarding ethics and feasibility for cross-specialty case acquisition. Our study covers 12 medical specialties. Using real patient records would require ethical approval from multiple medical institutions (to protect patient privacy). Additionally, electronic medical record systems across institutions have inconsistent formats. In contrast, cases derived from PubMed literature adhere to international standardized case reporting guidelines, ensuring highly structured information. These cases also undergo ethical review prior to publication, enabling the rapidly standardized collection of cross-specialty cases and guaranteeing the study’s feasibility. Second, alignment with the evaluation focus. The objective of this study is to identify LLMs’ performance differences under different specialty knowledge systems. While literature-derived cases lack dynamic information via real-time patient communication, they already include the core clinical decision-making elements of each specialty (e.g., specialty-specific symptoms, key test indicators), which are sufficient to support the objective of identifying cross-specialty differences. This conclusion is essentially independent of whether cases come from real records. To minimize bias from literature-derived cases, we manually excluded extremely rare cases to align the case spectrum as closely as possible with common clinical scenarios.

## Conclusion

This study finds significant disparities in LLMs’ performance across medical specialties. GPT-4o outperforms GPT-3.5-Turbo and Claude-3-Sonnet consistently, showing better stability in diagnostic and therapeutic conditions, while the latter two have more notable limitations, especially in cross-specialty diagnosis and dynamically integrating clinical guidelines. By leveraging LLMs across the full care continuum - from initial diagnosis to definitive therapy - this work charts a roadmap for AI-augmented clinical decision-making, with a focus on addressing performance disparities across specialties, enhancing multimodal reasoning (especially with medical imaging), and enabling dynamic guideline integration. This advances medical practice toward reliable, guideline-aligned treatment recommendations and personalized patient management.

## Supplementary Information

Below is the link to the electronic supplementary material.

Supplementary Material 1

## Data Availability

The data included during the current study are available from the corresponding author on reasonable request.

## Abbreviations

AI: Artificial intelligence
LLMs: Large language models
SD: Standard deviation
