# Supplementary material for: Performance of large language model in cross-specialty medical scenarios
Source: J Transl Med. 2025 Dec 22;24:211. doi: 10.1186/s12967-025-07577-x (PMC12903388; doi:10.1186/s12967-025-07577-x)
Supplement: Supplementary file 1 — Supplementary Material 1 [file 12967_2025_7577_MOESM1_ESM.docx]

**Supplemental Material**

**Performance of Large Language Model in Cross-Specialty Medical Scenarios**

Zhen Cui^1^; Wuzheng Liu^1^; Xuan Tian^2^; Conglei You^3^; Xiangyu Meng^4^; Huijuan Zhang^5^; Kangzi Gong^6^; Xu Wang^1^; Jun Wu^1^

**Correspondence:**

Jun Wu

Email: wujunpostbox@sina.com

**Supplement Material 1. Table S1.** Prompts and Responses related to a Clinical Case Vignette.

**Table S2.** A Representative Clinical Case of Low-Scoring LLMs’ Response to Therapeutic Recommendations.

**Table S1**. Prompts and Responses related to a Clinical Case Vignette.

| **Diagnostic Vignette** |
| --- |
| **History of Present Illness**  A 20-year-old woman presented to the emergency department with a 1-week history of headache and a 1-day history of double vision and drooping of the right eyelid.  **Past Medical History**  She had received a diagnosis of influenza 1 week before presentation. |
| **Laboratory** |
| Blood cultures grew *Fusobacterium* *necrophorum*.  **Radiology**  Contrast-enhanced magnetic resonance imaging of the head showed cavernous sinus thrombosis on both sides (Panel B, red arrows) with compression of the right oculomotor nerve (Panel B, yellow arrow). Computed tomographic venography of the head and neck showed thrombi in both internal jugular veins (Panel C, red arrows). Chest imaging showed no signs of septic emboli. |
|  |
| **Question** |
| Based on the provided patient information, and medical history, generate a precise diagnostic analysis. Focus on the most likely diagnosis with evidence-based rationale. Provide detailed, clinically actionable explanations. |
| **The diagnosis and treatment recommendations of LLMs.** |
| **ChatGPT-4o (score 10, 10)** |
| Based on the provided medical records, the symptoms and examination findings of this 20-year-old female patient suggest a diagnosis of Lemierre’s syndrome caused by Fusobacterium necrophorum infection.  Diagnostic Analysis and Rationale:  Symptoms and Signs:  The patient presents with symptoms such as headache, double vision, and right ptosis, indicating neurological involvement, particularly of the oculomotor nerve (third cranial nerve).  Physical examination reveals right ptosis, downward and outward deviation of the eyeball, and mydriasis (dial nonreactivity), which are consistent with oculomotor nerve palsy.  Imaging Studies:  Magnetic resonance imaging (MRI) reveals thrombosis in the cavernous sinuses bilaterally, with compression of the right oculomotor nerve.  Computed tomography venography (CTV) demonstrates thrombosis in the internal jugular veins bilaterally.  Etiological Evidence:  Blood culture results show Fusobacterium necrophorum, an anaerobic bacterium often associated with Lemierre’s syndrome.  Additional Test Results:  Chest imaging shows no signs of septic emboli, ruling out the spread of a pulmonary infection.  Diagnostic Explanation:  Lemierre’s syndrome is a suppurative and thrombotic phlebitis syndrome caused by an oropharyngeal infection (typically pharyngitis or tonsillitis), with the most common pathogen being Fusobacterium necrophorum. The syndrome is characterized by the formation of thrombosis in the internal jugular veins and secondary septicemia. Patients may present with symptoms following an upper respiratory tract infection, which can rapidly progress to systemic infection and multiple thrombi in various locations. |
| Therapeutic Recommendations:   1. Antibiotic Therapy:   Administer antibiotics that are effective against anaerobic bacteria, such as penicillins or cephalosporins, which may be used in combination with metronidazole or similar agents.   1. Anticoagulant Therapy:   Consider the use of anticoagulant medications to prevent further progression of the thrombus.   1. Close Monitoring:   Vigilantly monitor for signs of increased intracranial pressure, neurological function, and dissemination of infection.  In this scenario, timely administration of antibacterial therapy is crucial for prognosis and should be initiated without delay. |
| **Claude-3-Sonnet (score 8, 5)** |
| Based on the medical history and examination findings, the most likely diagnosis for this 20-year-old female patient is cavernous sinus thrombosis. Here is the detailed analysis:  Symptoms and Signs:  The patient exhibits symptoms such as headache, double vision, right ptosis (eyelid drooping), right eye medial and downward deviation, fixed pupil size, and inability to internally rotate the eye upward or downward, which are consistent with the presentation of cavernous sinus compression and oculomotor nerve palsy.  Imaging Studies:  a) Contrast-enhanced magnetic resonance imaging (MRI) reveals thrombosis in the cavernous sinuses bilaterally (Panel B, red arrows), with compression of the right third cranial nerve (Panel B, yellow arrows).  b) Computed tomography angiography (CTA) demonstrates thrombosis in the internal jugular veins bilaterally (Panel C, red arrows).  Etiological Examination:  Blood culture identifies Fusobacterium necrophorum, a bacterium commonly associated with oral and head and neck infections that can lead to cavernous sinus thrombosis.  Clinical Context:  The patient was diagnosed with influenza a week prior, which may have precipitated a secondary infection.  Integrating the symptoms, signs, imaging, and laboratory results, cavernous sinus thrombosis is the diagnosis that best fits the patient's profile. Cavernous sinus thrombosis typically results from the spread of local infections and can lead to cranial nerve diseases that are life-threatening.  Therapeutic Recommendations:  This patient requires immediate anticoagulant therapy, antibiotic treatment, and possibly thrombectomy if necessary, to prevent complications from worsening. |
| **GPT-3.5-Turbo (score 8, 5)** |
| Based on the medical history and examination results provided in the attachment, the 20-year-old female patient presented with symptoms such as headache, binocular vision issues, and right ptosis. According to her medical history, she was diagnosed with influenza a week prior to her current visit. Physical examination revealed right ptosis, enophthalmos (sunken eye), and lateral deviation of the right globe with poor pupillary light reflex, and restricted ocular motility. Magnetic resonance angiography (MRA) of the head demonstrated thrombosis within the cavernous sinuses bilaterally, with compression of the right oculomotor nerve. Cervical computed tomography venography (CTV) revealed thrombosis in the internal jugular veins bilaterally. Blood culture tested positive for Fusobacterium necrophorum, and chest imaging did not show signs of septic emboli.  Given this information, the patient’s clinical presentation and examination findings are consistent with cavernous sinus thrombosis (CST). The cavernous sinuses are significant venous sinuses located at the base of the skull, and their infectious thrombosis is typically caused by bacterial infections, potentially related to the preceding influenza. This condition can cause ocular symptoms (such as ocular motility disturbances and pupillary abnormalities), headache, and other symptoms. Additionally, the presence of thrombosis in the internal jugular veins bilaterally may be associated with the cavernous sinus thrombosis.  Therapeutic Recommendations:  In terms of treatment, antibiotics are the primary modality to cover pathogens such as Fusobacterium, and anticoagulant therapy may also be indicated. Additionally, it is essential to closely monitor the patient’s condition and complications, including intracranial infections. This represents a serious medical condition requiring prompt intervention and management. It is advised to immediately consult a neurologist or an infectious disease specialist for further evaluation and treatment. |
|  |

**Table S2.** A Representative Clinical Case of Low-Scoring LLMs’ Response to Therapeutic Recommendations.

| **Therapeutic Vignette** |
| --- |
| **History of Present Illness**  A 62-year-old woman presented to our hospital with a 2-hour history of abrupt-onset of right flank pain and vomiting.  **Past Medical History**  The patient had a history of vertigo and recent unintentional weight loss with migrating lymphadenopathy. |
| **Laboratory** |
| Urine analysis showed 1+ protein, positive leukocyte esterase; microscopic examination showed 12 white blood cells, and 8 red blood cells. The patient to be anaemic with a haemoglobin concentration of 6.8 g/dL (reference range 11.7–15.7). Further tests showed a white blood cell count of 49 × 10³ per µL (reference range 4.0–11.0), platelets 380 × 10³ per µL (reference range 150–450), and serum creatinine concentration of 0.9 mg/dL (reference range 0.51–0.95).  Over the next 2 weeks, analysis of a sample obtained by bone marrow biopsy showed monocytosis—including atypical monocytes and slight dysgranulopoiesis; G-banded chromosomal analysis showed a 46, XX karyotype and genetic sequencing for common myeloid neoplasm associated gene mutations showed CBL R420*, CBL C382S, IDH2 R140Q, and SRSF2 P95R mutations.  **Radiology**  Contrast-enhanced magnetic resonance imaging of the head showed cavernous sinus thrombosis on both sides with compression of the right oculomotor nerve. Computed tomographic venography of the head and neck showed thrombi in both internal jugular veins. Chest imaging showed no signs of septic emboli. |
| **Question** |
| Based on the provided patient information, and medical history, generate a precise diagnostic analysis. Focus on the most likely diagnosis with evidence-based rationale. Provide detailed, clinically actionable explanations. |
| **The therapeutic recommendations of LLMs.** |
| **ChatGPT-4o (score 0)** |
| Based on the provided medical records, the patient may have acute myeloid leukemia (AML) or another myeloproliferative disorder.  Further genetic testing and bone marrow biopsy results will facilitate a definitive diagnosis and guide treatment planning. Close monitoring of the patient’s blood parameters is essential, along with proactive management of complications such as renal insufficiency and spontaneous bleeding. |
| **Claude-3-Sonnet (score 0)**  Based on the medical records and test results, this 62-year-old female patient’s primary diagnosis is acute myeloid leukemia.  Acute leukemia can induce a bleeding tendency and infiltrate multiple organs (e.g., kidneys, spleen). Prompt and comprehensive diagnosis and treatment are critical for this case. Standard induction chemotherapy for AML should be initiated immediately, along with aggressive management of complications such as hemorrhage and renal failure. In summary, this represents a typical clinical presentation of acute myeloid leukemia with a definitive diagnosis. |
| **GPT-3.5-Turbo (score 0)**  Based on the patient’s medical history and test results, the following diagnostic considerations are proposed: Wunderlich syndrome, Leukemia, Other myeloid disorders.  Further clinical examinations and evaluations, including in-depth bone marrow pathological analysis, molecular genetic testing, and comprehensive tumor marker screening, will facilitate the confirmation of a definitive diagnosis. Collaboration among a multidisciplinary team, including hematologists, nephrologists, and surgeons, is crucial for formulating a comprehensive diagnosis and treatment plan.  In summary, based on the patient’s medical history and test results, additional assessments and diagnostic workup are required to determine the final diagnosis and develop a corresponding treatment strategy. |
